# Editorial for Special Issue: Approaches to Top-Down Proteomics: In Honour of Prof. Patrick H. O’Farrell

**DOI:** 10.3390/proteomes5030018

**Published:** 2017-07-24

**Authors:** Jens Coorssen, Alfred Yergey

**Affiliations:** 1 Faculty of Graduate Studies, and the Departments of Health Sciences and Biological Sciences, Brock University, St. Catharines, L2S 3A1 ON, Canada; 2 NIH, Building 10, Room 9D 52, Bethesda, MD 20892, USA

Presaging the current discipline of Proteomics, Prof Patrick H. O’Farrell recognized the critical need for detailed protein analyses to dissect and thereby understand molecular mechanisms. His development of two-dimensional gel electrophoresis (2DE) has proven a cornerstone of modern protein science and, together with developments in mass spectrometry and genomics, laid the groundwork for current Systems Biology approaches to understanding the molecular basis of physiological states in health and disease. Notably, as refined over the last 40 years, 2DE provides the only currently available, optimized, routine, top-down analytical approach that resolves proteoforms (i.e., protein species including isoforms and post-translationally modified variants)—those protein entities that define biological functions. In this sense, this issue is directed more towards the somewhat philosophical concept of characterizing a protein, in all of its variations or species, but does not focus particularly on the peptide-centric approaches that have become so common in the realm of mass spectrometry-centered “proteomics”. As a consequence of this philosophical distinction, this issue attempts to avoid the rather controversial debate on particular instrumentation, but rather tries to move toward the ideal of employing fitness-for-purpose as the means for deciding what instrumentation should be used to address the questions required to characterize a complete proteome and its inherent proteoforms, as much as possible, preferably in its entirety.

This Special Issue of Proteomes—Approaches to Top-Down Proteomics—is thus in recognition of Patrick O’Farrell’s contributions, providing a sampling of the current state-of-the-art as well as future directions. To serve as both a teaching tool and analytical resource for students through to experienced researchers, we sought both a review of existing methods as well as how these are being applied, refined, and further developed for future applications. We believe this approach will revise much of the dogma that has arisen in the literature over the last 20+ years with regard to top-down proteomics, and to illuminate promising directions in the further dissection and understanding of molecular mechanisms and thus cellular processes, as well as the identification of biomarkers.

The content of this Special Issue can be divided under two very general headings: Established Methods and Applications, and New Directions. Padula et al. [1]. provide a detailed review and evaluation of 2D-PAGE as a top-down analytical approach relative to bottom-up, providing critical insights into the current status of proteome analyses, noting that continued methodological evolution in conjunction with MS is the pathway to eventually enabling the comprehensive profiling of proteomes. In a complementary paper, Pergrande and Cologna [2] provide a critical review of isoelectric focusing and derivative approaches, noting that a move to dealing with ever smaller samples holds much future promise in light of the outstanding advances of the last four decades. Recognizing the central role of MS in enabling all proteomic analyses, Vyatkina [3] reviews the current state of de novo sequencing by tandem MS, and enhancements to the Twister algorithm that enable the sequencing of longer fragments of a given protein. Considering the power of MS in driving current proteomic approaches, Naryzhny [4] reviews the historical developments that have yielded continuous improvements in detection sensitivity and proposes a next step, what might be termed a large-scale ‘2DE-shotgun MS/bioinformatics’ approach, in order to extract as much peptide information as possible from high resolution gel-based separations. 

With a focus on post-translational modifications, Kusch et al. [5] present a new partial immunoblotting approach coupling 2DE with the detection of specific molecular alterations, enabling a direct coupling with MS confirmation of the protein identification; in this method, there are promising potential clinical applications. Murphy et al. [6] review the extensive contributions of top-down proteomics to understanding muscle development, physiology, and aging. D’Silva et al. [7] test and refine a number of approaches from the literature finding that established fractionation techniques are not consistent with the need for quantitative analyses; the result is a routine 2DE approach to serum analysis that seeks to embrace the complexity of the native sample in order to best enable the quantitative identification of potential biomarkers. There is promise in this routine approach to identify much needed biomarkers from blood and other fluids.

Baraúna et al., and Campos and Almeida [8,9] provide reviews and perspectives on bacterial cold adaptation and livestock and aquatic sciences, respectively. The former focuses in particular on the integration of broader omics datasets, while the latter emphasizes the important role of proteomics in the area of farm animal health and thus food quality.

With reference to new and developing directions, Raykova et al. [10] review the pros and cons of different microscopy-based, single-cell molecular analyses and their application to assessing tissues in health and disease. In a comparable vein, Li et al. [11] present a signal amplification strategy to selectively assess target proteins within a complex molecular milieu using aptamer-functionalized gold nanoparticles. 

The future clearly holds even more promise in terms of the further development of both discovery and targeted top-down proteomics to understanding biological pathways and mechanisms. Available tools and techniques from both top-down and bottom-up approaches should be considered complementary and used as such to address many of our outstanding and growing health and environmental concerns. 
